# Association of Immune Thrombocytopenia and Celiac Disease in Children: A Retrospective Case Control Study

**DOI:** 10.4274/tjh.galenos.2021.2021.0128

**Published:** 2021-08-25

**Authors:** Angela Guarina, Maddalena Marinoni, Giuseppe Lassandro, Paola Saracco, Silverio Perrotta, Elena Facchini, Lucia Dora Notarangelo, Giovanna Russo, Paola Giordano, Francesca Romano, Elisa Bertoni, Chiara Gorio, Gianluca Boscarol, Milena Motta, Marco Spinelli, Angelica Barone, Marco Zecca, Francesca Compagno, Saverio Ladogana, Angela Maggio, Maurizio Miano, Gianluca Dell’Orso, Elena Chiocca, Ilaria Fotzi, Angela Petrone, Assunta Tornesello, Irene D’Alba, Silvia Salvatore, Maddalena Casale, Giuseppe Puccio, Ugo Ramenghi, Piero Farruggia

**Affiliations:** 1 U.O.C. Oncoematologia Pediatrica, ARNAS Civico, Di Cristina, Benfratelli, Palermo, Italy; 2 Pediatria-DH Oncoematologico Pediatrico, SSD Oncoematologia Pediatrica-Ospedale Filippo Del Ponte, Varese ASST Settelaghi, Varese, Italy; 3 Department of Biomedical Sciences and Human Oncology, University of Bari “Aldo Moro,” Bari, Italy; 4 SC Pediatria Specialistica Universitaria, AOU Città della Salute e della Scienza, Presidio Ospedale Infantile Regina Margherita, Torin, Italy; 5 U.O. S.D. Ematologia e Oncologia Pediatrica Dai Materno Infantile - Università degli Studi della Campania Luigi Vanvitelli, Naples, Italy; 6 Clinica Pediatrica Oncologia Ed Ematologia Pediatrica “Lalla Seràgnoli - Policlinico Sant’Orsola Malpighi,” Bologna, Italy; 7 U.O. Oncoematologia Pediatrica, Presidio Ospedale dei Bambini, Spedali Civili, Brescia, Italy; 8 UOC Ematologia ed Oncologia Pediatrica con TMO - AOU Policlinico “Rodolico-San Marco,” Università di Catania, Catania, Italy; 9 Ospedale Regionale Dipartimento di Pediatria, Bolzano, Italy; 10 Fondazione MBBM/AO San Gerardo Clinica Pediatrica Universitaria, Monza, Italy; 11 U.O. Pediatria e Oncoematologia - AOU, Parma, Italy; 12 SC Oncoematologia Pediatrica - Fondazione IRCCS, Policlinico San Matteo, Pavia, Italy; 13 UOC Oncoematologia Pediatrica - IRCCS Ospedale Casa Sollievo della Sofferenza, San Giovanni Rotondo, Italy; 14 Dipartimento di Scienze Pediatriche Generali e Specialistiche, U.O.C. Oncologia IRCCS Istituto Giannina Gaslini, Genoa, Italy; 15 Oncologia, Ematologia e TCSE - Centro di Eccellenza di Oncologia ed Ematologia - AOU A. Mayer, Firenze, Italy; 16 U.O.M. Pediatria Ospedale S. Chiara, Trento, Italy; 17 U.O.C. Di Oncoematologia Pediatrica - PO “Vito Fazzi”, Lecce, Italy; 18 S.O.S.D., Oncomematologia Pediatrica, A.O.U. Azienda Ospedali Riuniti, Ospedale Pediatrico Salesi, Ancona, Italy; 19 Dipartimento di Pediatria, Università degli Studi dell’Insubria, Varese, Italy

**Keywords:** Celiac, Children, Immune, Thrombocytopenia, Pediatric

## Abstract

**Objective:**

The association between celiac disease (CD) and immune thrombocytopenia (ITP) is still uncertain. The aim of this study was to characterize the coexistence of these two diseases in Italian children.

**Materials and Methods:**

This is a retrospective multicenter study investigating the occurrence of CD in 28 children with ITP diagnosed from January 1, 2000, to December 31, 2019.

**Results:**

The first diagnosis was ITP in 57.1% and CD in 32.1% of patients. In 3 patients (10.7%), the two diagnoses were simultaneous. All the potential and silent cases of CD in our cohort were diagnosed in the groups of “ITP first” and “simultaneous diagnosis”. In all children ITP was mild, and in 2 out of 8 not recovered from ITP at the time of CD diagnosis a normalization of platelet counts (>100,000/μL) occurred 3 and 5 months after starting a gluten-free diet, respectively.

**Conclusion:**

We think that screening for CD should be considered in children with ITP regardless of the presence of gastrointestinal symptoms. Furthermore, some patients may recover from ITP after starting a gluten-free diet.

## Abstract (tr)

**Amaç:**

İmmün trombositopeni (İTP) ve çölyak hastalığı (ÇH) arasındaki ilişki hala belirsizliğini korumaktadır. Bu çalışmanın amacı İtalyan çocuklarında bu iki hastalık arasındaki ilişkiyi ortaya koymaktır.

**Gereç ve Yöntemler:**

Bu çalışma, 1 Ocak 2000’den 31 Aralık 2019’a kadar İTP tanısına ek olarak ÇH tanısı alan 28 çocuğun araştırıldığı geriye dönük çok merkezli bir çalışmadır.

**Bulgular:**

Hastaların %57,1’inde ilk tanı İTP ve %32,1’inde ÇH idi. Üç hastada (%10,7) iki tanı aynı anda konulmuştu. Kohortumuzdaki tüm potansiyel ve sessiz ÇH olguları “önce İTP” ve “eşzamanlı tanı” gruplarında teşhis edildi. Tüm çocuklarda İTP hafifti ve ÇH tanısı sırasında trombositopenisi devam eden 8 çocuktan 2’sinde, glutensiz diyete başladıktan sonra sırasıyla 3 ve 5 ayda trombosit sayılarında normalleşme (>100.000/μL) görüldü.

**Sonuç:**

Bu çalışma İTP’li çocuklarda gastrointestinal semptomların varlığına bakılmaksızın ÇH taramasının yapılması gerektiğini düşündürmektedir. Bazı hastalarda trombosit sayılarının glutensiz bir diyete başladıktan sonra düzelebileceği akılda bulundurulmalıdır.

## Introduction

Celiac disease (CD) is an immune disorder of the small intestine, often associated with other autoimmune disorders [1], whose prevalence has been estimated in Europe as 0.7% [2]. Immune thrombocytopenia (ITP) is an acquired disease caused by autoantibodies against platelet antigens whose estimated yearly incidence in the pediatric population is 0.0019%-0.0064% [3]. The features of ITP and CD coexistence have been investigated in some cohorts and case reports with contradictory results, especially regarding the efficacy of a gluten-free diet on ITP [4,5,6,7,8,9,10,11,12,13,14,15,16,17,18]. Furthermore, no study has aimed to describe the specific features of this association in the pediatric population. The goal of this study was to characterize the association of these two diseases in Italian children.

## Materials and Methods

A case report form (CRF) was sent to all 55 AIEOP (Associazione Italiana Emato-Oncologia Pediatrica) Centers collecting information about patients up to 18 years of age with both CD and ITP diagnosed from January 1, 2000, to December 31, 2019. Children affected by a known immunodeficiency were excluded. The clinical phenotypes of ITP and CD autoantibody testing (anti-transglutaminase antibodies ± anti-endomysial antibodies) ± biopsy results were recorded. CD was considered and classified according to Tonutti and Bizzaro [19] as classic (presence of symptoms of malabsorption, positive CD-specific antibodies and biopsy, and symptom resolution with a gluten-free diet), silent (positive CD-specific antibodies, HLA DQ2 or DQ8, and biopsy without symptoms), latent (presence of HLA DQ2 or DQ8 and histological alterations typical of CD in duodenal biopsy at some point in life, but neither symptoms nor positive antibodies), or potential (positive CD-specific antibodies and HLA without histological abnormalities in duodenal biopsies). ITP was defined as newly diagnosed (within 3 months from diagnosis), persistent (between 3 and 12 months from diagnosis), or chronic (cITP, lasting for more than 12 months) [20]. The ITP bleeding score was defined as follows [21]: Type A: asymptomatic-paucisymptomatic ITP, with clinical symptoms ranging from no bleeding to a few petechiae and some bruises without mucosal hemorrhages; Type B: intermediate ITP, a clinical picture with more petechiae, bruising, and mucosal hemorrhages; Type C: severe ITP, a clinical picture with severe cutaneous and mucosal bleeding symptoms with at least one of the following: retinal hemorrhages, intracranial hemorrhage, other severe internal hemorrhages, hemorrhagic shock, or life-threatening bleeding. The timing of the diagnosis of the patients was classified as follows: 1) CD first: CD was diagnosed before ITP; 2) ITP first: ITP was diagnosed before CD; 3) Simultaneous diagnosis: the second disease (CD or ITP) was diagnosed during hospitalization or initial ascertainment for the other disorder. Considering therapy or efficacy of a gluten-free diet on ITP, complete response was defined as any platelet count of ≥100,000/µL with the absence of bleeding, partial response as any platelet count between 30,000 and 100,000/µL with at least doubling of the baseline count and absence of bleeding, and no response as any platelet count of <30,000/µL or less than doubling of the baseline count and/or occurrence of bleeding.

### Statistical Analysis

Statistical analysis was performed using open source R statistical software [22].

## Results

We collected CRFs for 28 ITP/CD patients diagnosed in Italy from 2000 to 2019; they were all Caucasian (male/female=1/2.1). Among CD patients, for 11 the diagnosis was performed through intestinal biopsy + anti-transglutaminase ± endomysial antibodies detection, and for the remaining 17 through antibody dosages only according to ESPGHAN guidelines. The most relevant clinical features of the cohort are shown in Table 1. The median follow-up (FUP) time from ITP and CD diagnosis was 4.4 years (range: 0.2-16.5) and 2.3 years (range: 0.02-15.2), respectively. Median age at CD and ITP diagnosis was 6.2 and 5.8 years, respectively. Among 27 evaluable patients, a positive family history for other autoimmune diseases was present in 15 (55.6%): thyroid disorders were reported in 66.6% of these cases and CD in 40%. ITP was present in the family history of 1 patient only (grandfather). Extraintestinal disorders affected 4 patients (14.8%); interestingly, 3 out of 4 were autoimmune hemolytic anemia. The median number of platelets at ITP diagnosis was 19,500/µL (range: 1,000-96,000) and the bleeding score was A in 67.9% and B in 32.1% of patients, respectively. First-line therapy for ITP was IVIG in 18 patients (64.3%) and oral prednisone in 4 patients (14.3%), while 6 patients (21.4%) were not treated (“wait and see”). ITP turned out to be persistent in 12 out of 27 evaluable cases (44.4%) and chronic in 10 out of 25 evaluable cases (40%); the median number of platelets at 3 and 12 months from ITP onset was 100,000/µL (range: 1,000-446,000) and 135,000/µL (range: 15,000-343,000), respectively; only 1 patient at 12 months from ITP diagnosis had platelets of >20,000/µL. At the last FUP, 25.0% of patients were still presenting with cITP; none of them had platelets of >50,000/µL and only 1 patient was still on treatment (with mycophenolate mofetil). *Helicobacter pylori* was tested in 57.1% of patients using a stool antigen test and was found negative in all cases. Bone marrow aspiration was performed for 57.1% of patients and was always consistent with ITP. CD was classified as classical, silent, latent, and potential in 17 (60.7%), 7 (25%), 2 (7.1%), and 2 (7.1%) cases, respectively. The first diagnosis was ITP for 16 patients (57.1%) and CD for 9 patients (32.1%). For 3 patients (10.7%), the two diagnoses were simultaneous; 1 potential and 2 classical cases of CD were discovered just after ITP onset, in 1 patient after having performed an autoimmune panel for screening and in the other 2 after having evaluated pre-existing gastrointestinal symptoms. The most relevant features of the “CD first” and “ITP first” patients are shown in Table 2. In the “CD first” subgroup (9 patients), the median time from diagnosis of CD to diagnosis of ITP was 1.6 years (range: 0.2-10.1). These 9 cases of CD were all of the classic type, but 2 (latent) patients were on a gluten-free diet at the moment of ITP diagnosis. None of them suffered from other autoimmune diseases and 3 out 8 evaluable patients (37.5%) developed cIPT. In the “ITP first” subgroup (16 patients), the median time from diagnosis of ITP to diagnosis of CD was 2.8 years (range: 0.1-12.9) and all cases of silent CD (n=7), diagnosed thanks to the autoimmune screening performed in the centers, were seen among these patients. At ITP onset 5 patients (31.2%) were not treated (“wait and see”), 9 (56.2%) received intravenous immunoglobulin (IVIG), and 2 (12.5%) received oral prednisone; after that, at the moment of CD diagnosis, 5 (31.2%) had not yet been treated, 5 (31.2%) had received IVIG, 5 (31.2%) had received both IVIG and oral prednisone, and 1 (6.2%) had received oral prednisone only.

Subsequent rescue therapies for ITP were attempted for 12 out of 28 patients (42.9%): half, relapsed after prednisone, were treated with IVIG and half, relapsed after IVIG, with oral prednisone. Only 6 patients (21.4%) needed further treatments: 3 children received IVIG (one of them was later treated with eltrombopag), 2 intravenous methylprednisolone, and 1 mycophenolate mofetil. In 2/9 (22.2%) “ITP first” patients not recovered from ITP at the time of CD diagnosis (after a previous transient response to both IVIG and oral prednisone), the gluten-free diet seemed to have played a role in ITP recovery. One child who had ITP lasting for 37 months showed an increase of platelets from 48,000/µL to 118,000/µL after 5 months of diet; in the second patient, whose ITP had lasted for 33 months, platelets increased from 13,000/µL to 107,000/µL after 3 months of a gluten-free diet. These patients had received the last ITP treatment 36 and 23 months before the start of the gluten-free diet, respectively.

## Discussion

ITP is a hematologic disease characterized by thrombocytopenia caused by antiplatelet autoantibodies that, binding to platelet membrane glycoproteins, mediate the destruction of platelets in the reticuloendothelial system, particularly in the spleen and liver [20,21,23]. The association of ITP with other autoimmune diseases, above all thyroid autoimmune diseases and systemic lupus erythematosus, is a well-recognized condition [23,24]. CD is an autoimmune disease that represents the most common life-long food-sensitive enteropathy in humans: it is characterized by malabsorption and villous atrophy occurring as a consequence of the ingestion of wheat gluten or related rye and barley proteins in genetically predisposed individuals [25]. A wide range of immune disorders [8,26,27,28,29] have been associated with CD with a prevalence that can be 7-fold higher than in healthy people [8], but little is known, particularly in the pediatric population, about the association between CD and ITP. Apart from the registry analysis of Olen et al. [4], only 16 pediatric patients have been reported, mostly in single case reports [6,8,9,10,11,12,13,14,15,16,17]. The ITP prevalence in children <18 years of age is 6/100,000 [30], and so in Italy in 2019, among roughly 55,000 CD patients of pediatric age [31], there were 3 expected children also affected by ITP. However, in our cohort, we found double that number of children (n=6). A large Swedish registery-based cohort study [4] reported an increased risk of ITP in CD patients [hazard ratio: 1.91, 95% confidence interval (CI): 1.19-3.11] and a positive association between CD and prior ITP (odds ratio: 2.96, 95% CI: 1.60-5.50). Our results are in accordance with the Swedish data [4], but not with the findings of Ventura et al. [8], who analyzed 909 people of all ages. However, it must be emphasized that the 2 patients only affected by ITP in that Italian cohort of 909 CD cases [4] were children and that the present study is the first to focus on patients of pediatric age, a period of life when a peak of ITP incidence is seen [32,33]. Based on the available data, it is impossible to explain the reasons for the probable higher incidence of ITP among pediatric CD patients. It can be speculated that ITP is secondary to immunostimulation from luminal antigens, but it is also possible to postulate a common underlying immune dysregulation similarly to what is observed in secondary ITP in the course of other autoimmune disorders such as lupus erythematosus or common variable immunodeficiency [34].

The median ages at diagnosis of CD (6.2 years) and ITP (5.8 years) in our cohort are in accordance with what has been previously reported [35]. The prevalence of female gender confirms what is observed in the vast majority of studies about CD [4,7,36], seeming to overcome the opposite slight prevalence of male gender observed in childhood ITP [37]. All the potential (n=2) and silent (n=7) cases of CD in our cohort were diagnosed in the groups of “ITP first” and “simultaneous diagnosis”, where CD was diagnosed during the first tests for ITP, raising the question of CD screening at ITP onset. In our opinion, despite conflicting results regarding the frequency of association for these 2 diseases, in view of the possible complications in untreated CD patients and the limited costs of anti-transglutaminase antibodies (30 euros in Italy) [38], screening for CD could be performed.

ITP features, irrespectively of their appearance happening before or after CD, are very similar in our cohort to what is typically observed in pediatric ITP, with mild onset and course [20,21]. No patient suffered from severe hemorrhage and only 1 was on treatment at the last FUP. Based on the limited data on the association of CD and ITP, the effect of a gluten-free diet is still uncertain, being beneficial in some cases [6,11,13,16] and having no impact in others [9,10,15]. In our analysis, the gluten-free diet seemed to be efficient in no more than 20%-25% of patients already affected by ITP.

### Study Limitations

The limitations of the present study are related to the inherent biases in a retrospective study. Furthermore, even though the majority of Italian AIEOP Centers perform autoimmunity screening including anti-transglutaminase autoantibodies, that screening is conducted with variable timing and it is sometimes omitted. Thus, it is possible that some cases of CD, especially cases that are not of the classic type, have been missed. At the same time, even with these limitations, the present work is the first study reporting clinical insights and detailed information on the ITP/CD association in an exclusively pediatric cohort.

## Conclusion

According to our data, it seems that ITP presents at a higher prevalence among pediatric CD patients. Therefore, at every ITP onset in otherwise healthy children, clinicians should be aware that an association with undiagnosed CD is possible. Finally, it has to be remarked that ITP in these patients presents the typical mild clinical features usually observed in childhood, and that after the diagnosis of CD, the start of a gluten-free diet could result in a significant increase of platelet levels in a few cases. However, future studies are needed to further explore the efficacy of diet in these children.

## Figures and Tables

**Table 1:**
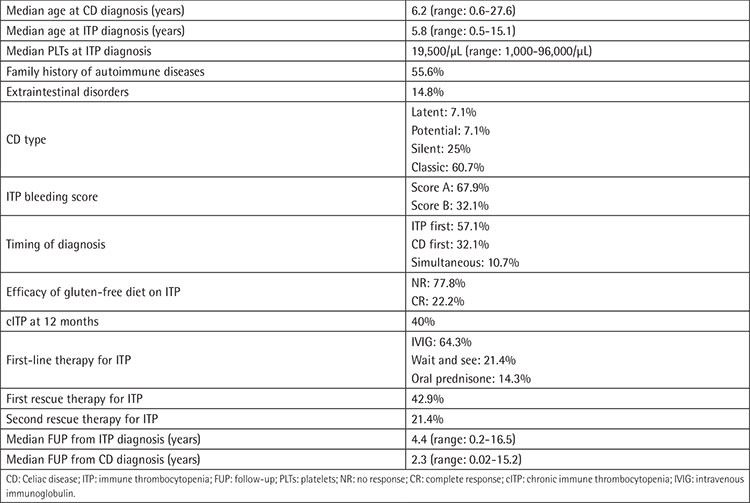
Characteristics of the patients.

**Table 2:**
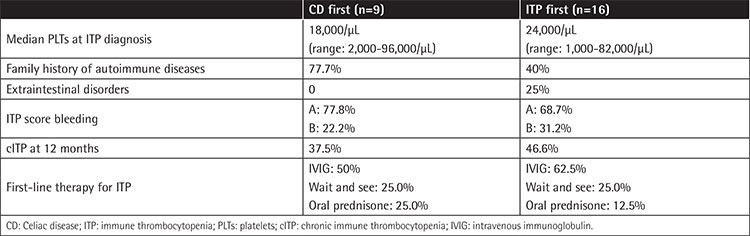
Characteristics of the CD first and ITP first patients.
